# Cancer-associated pyroptosis: A new license to kill tumor

**DOI:** 10.3389/fimmu.2023.1082165

**Published:** 2023-01-18

**Authors:** Qing Kong, Zhibin Zhang

**Affiliations:** Department of Immunology, The University of Texas MD Anderson Cancer Center, Houston, TX, United States

**Keywords:** pyroptosis, programmed cell death, killer lymphocyte, inflammasome, anti-tumor immunity, interleukin-1β, gasdermin

## Abstract

Pyroptosis is a programmed necrotic cell death mediated by pore-forming Gasdermin (GSDM) proteins. After being unleashed from the C-terminal auto-inhibitory domains by proteolytic cleavage, the N-terminal domains of GSDMs oligomerize and perforate on the plasma membrane to induce cytolytic pyroptosis, releasing immune mediators and alarming the immune system. Upon infection or danger signal perception, GSDMD that functions downstream of the inflammasome, a supramolecular complex for inflammatory caspase activation, is cleaved and activated by inflammasome-activated caspase-1/4/5/11 in immune cells and epithelial cells to trigger pyroptosis and exert anti-infection protection. Unlike this inflammasome-activated pyroptosis (IAP), recent studies also suggest an emerging role of cancer-associated pyroptosis (CAP), mediated by other GSDMs in cancer cells, in provoking anti-tumor immunity. IAP and CAP share common features like cell membrane rupture but also differ in occurrence sites, activating mechanisms, secreting cytokines and biological outcomes. Here we review the most recent knowledge of cancer-associated pyroptosis and present a promising avenue for developing therapeutic interventions to enhance anti-tumor immunity for cancer treatment.

## Introduction: Overview of Inflammasome-activated pyroptosis

Pyroptosis was first observed in 1986, when Friedlander reported that Anthrax lethal toxin induced rapid and massive cell death in mouse peritoneal macrophages (1). Similar observations were made by Zychlinsky and colleagues in macrophages challenged with *Shigella flexneri* and *Salmonella typhimurium* (2, 3), and their studies revealed that those pathogen-induced macrophage deaths featured caspase-1 activation and were accompanied by IL-1β and IL-18 release (2, 4), but these cell deaths were classified into apoptosis (2, 3). In 2000, Cookson and Brennan reported *Salmonella*-induced cell death as caspase-1-dependent necrosis (5), which exhibits distinct features compared to apoptosis. Pyroptosis displays giant membrane ballooning, while apoptosis exhibits cell shrinkage and small blebbing. In 2001, they named this proinflammatory cell death “pyroptosis”, in which the Greek root “pyro” means fire or fever, and “ptosis” denotes falling (6). Later studies confirmed that Anthrax lethal toxin treatment, *Shigella* challenge and *Salmonella* infection all induce pyroptosis in macrophages (7, 8). Thus, pyroptosis could be originally defined as a caspase-1-mediated inflammatory cell death triggered by sensing of invasive pathogens and danger signals in macrophages. Later on, this pyroptosis was also reported in other immune and non-immune cells, such as epithelial cells and cardiomyocytes.

This classical caspase-1-dependent pyroptosis is mediated by inflammasome, manifesting IL-1β/IL-18 secretion and cell membrane rupture. The inflammasome is a multi-protein platform responsible for caspase-1 activation, proposed by Tschop and coworkers in 2002 (9). Recognition of pathogen-associated molecular patterns (PAMPs) or danger-associated molecular patterns (DAMPs) by cytosolic pattern recognition receptors (PRRs) initiate the assembly of inflammasome, during which the PRR recruits an adaptor protein called ASC (Apoptosis-associated speck-like protein containing a CARD) as well as pro-caspase-1 to form a supramolecular complex, the inflammasome. ASC contains a pyrin domain (PYD) and a caspase recruitment domain (CARD), bridging the upstream PYD-containing PRR receptor and downstream CARD-containing caspase-1. The proximity-induced auto-processing of pro-caspase-1 leads to its activation, and active caspase-1 further maturates IL-1β and IL-18, and triggers pyroptosis (10). The most studied inflammasomes include but are not limited to NLRP1b inflammasome that is activated by lethal toxin (11, 12), AIM2 inflammasome for cytoplasmic double-strand DNA recognition (13–15), NLRP3 inflammasome that detects multiple danger signal molecules such as crystalline material, extracellular ATP and pore-forming toxins (16, 17), NLRC4 inflammasome that recognizes bacterial flagellin or type III secretion system components (18, 19), and Pyrin inflammasome that monitors the modification or inactivation of Rho GTPases by pathogens (20).

In addition to caspase-1-dependent canonical inflammasomes, a non-canonical inflammasome pathway has recently been identified (21–23), in which human caspase-4/5 and mouse caspase-11 act as cytosolic receptors for lipopolysaccharides (LPS) (24), a cell wall component from Gram-negative bacteria. Upon LPS binding, pro-caspase-4/5/11 oligomerize and are auto-activated to trigger pyroptosis. Although caspase-4/5/11 do not process IL-1β or IL-18, secretion of those cytokines are also detected during non-canonical inflammasome activation, which could be attributed to the secondary activation of NLRP3 inflammasome (25–27). The extensive studies on inflammasome pathways have been reviewed in detail (10, 28) and will not be further discussed here.

Inflammasome-activated pyroptosis (IAP) is a primary defense mechanism against intracellular bacterial infections (29). During lytic pyroptosis, intracellular pathogens, immune alarmins and other cellular components are released from the dead cells. Pyroptosis promotes the clearance of bacteria by disrupting the bacterial intracellular replication niche (30), directly damaging or eliminating the bacteria (31, 32), and enhancing host immune responses such as neutrophil-mediated bacterial killing (30, 31). Nonetheless, the molecular mechanism underlying pyroptosis remained unknown for decades until the seminal discovery of gasdermin D (GSDMD) in 2015.

## Identification of Gasdermins: The pyroptosis executioners

Through unbiased genome-wide CRISPR-Cas9 screens and a forward genetic screen with ethyl-*N*-nitrosourea-mutagenized mice, respectively, Shao and Dixit group independently identified gasdermin D (GSDMD) as the executor of pyroptosis in 2015 (33, 34). GSDMD is cleaved after D276 by inflammatory caspase-1/4/5/11, and the resulting GSDMD N-terminal (NT) fragment is required for pyroptosis. Later on, Shao, Lieberman and other groups discovered that GSDMD-NT induces pyroptosis by forming membrane pores (32, 35–38). GSDMD consists of a pore-forming N-terminal domain (GSDM-NT) and an autoinhibitory C-terminal domain (GSDM-CT), connected by a flexible linker. The cleavage of GSDMD in the linker by inflammatory caspases unleashes GSDM-NTs, which oligomerize and perforate on the plasma membrane, leading to the disruption of the plasma membrane and pyroptosis. The structure of the GSDMD-NT membrane pore further validated these discoveries (39).

Human GSDMD belongs to a gasdermin family of six proteins, namely GSDM A-D, GSDME (a.k.a. DFNA5) and DFNB59 (40). Gasdermin A was initially named in 2000 based on its selective expression in the gastrointestinal tract and dermis (41). In mice, there are no *GSDMB* counterpart but 3 *gsdmas* (*gsdma* 1-3) and 4 *gsdmcs* (*gsdmc 1-4*). GSDM A-E exhibit pore-forming activity because overexpressing the NT domains of those GSDMs trigger pyroptosis in HEK293T cells (36). GSDM-mediated pyroptotic cell death can be triggered by proteases other than inflammatory caspases, including apoptotic caspases, serine protease granzymes secreted by cytotoxic lymphocytes, proteases from neutrophil granules and pathogens (40). For instance, cysteine protease SpeB from *Streptococcus pyogenes* cuts GSDMA to induce pyroptosis in keratinocytes (42, 43). GSDMB can be cut and activated by GzmA (44). Both GSDMC and GSDMD could be cleaved and activated by caspase-8 (45–47), and GSDMD is also cleaved by neutrophil elastase (48, 49) and cathepsin G (50), which are implicated in neutrophil pyroptosis (netosis). GSDME is processed and activated by both Caspase-3 (51, 52) and GzmB (53, 54). More details can be found in other reviews (40). All those proteolytic cleavages similarly release the pore-forming GSDM-NTs to activate pyroptotic cell death. Therefore, pyroptosis has been redefined as gasdermin-mediated programmed necrotic cell death (55, 56), which means any cells expressing GSDMs could undergo pyroptosis, no matter through what activating mechanisms. From another perspective, GSDMs act as intracellular sensors of mislocalized cytosolic proteases. If considering SpeB as a PAMP from bacteria and Gzms as DAMPs from within, the GSDMs function as unconventional PRRs that sense danger-associated proteases and alarm the immune system by triggering pyroptosis. It will also be interesting to investigate whether GSDMs could be activated through mechanisms other than proteolytic cleavage.

## The cancer-associated pyroptosis in tumors

**Figure 1:**
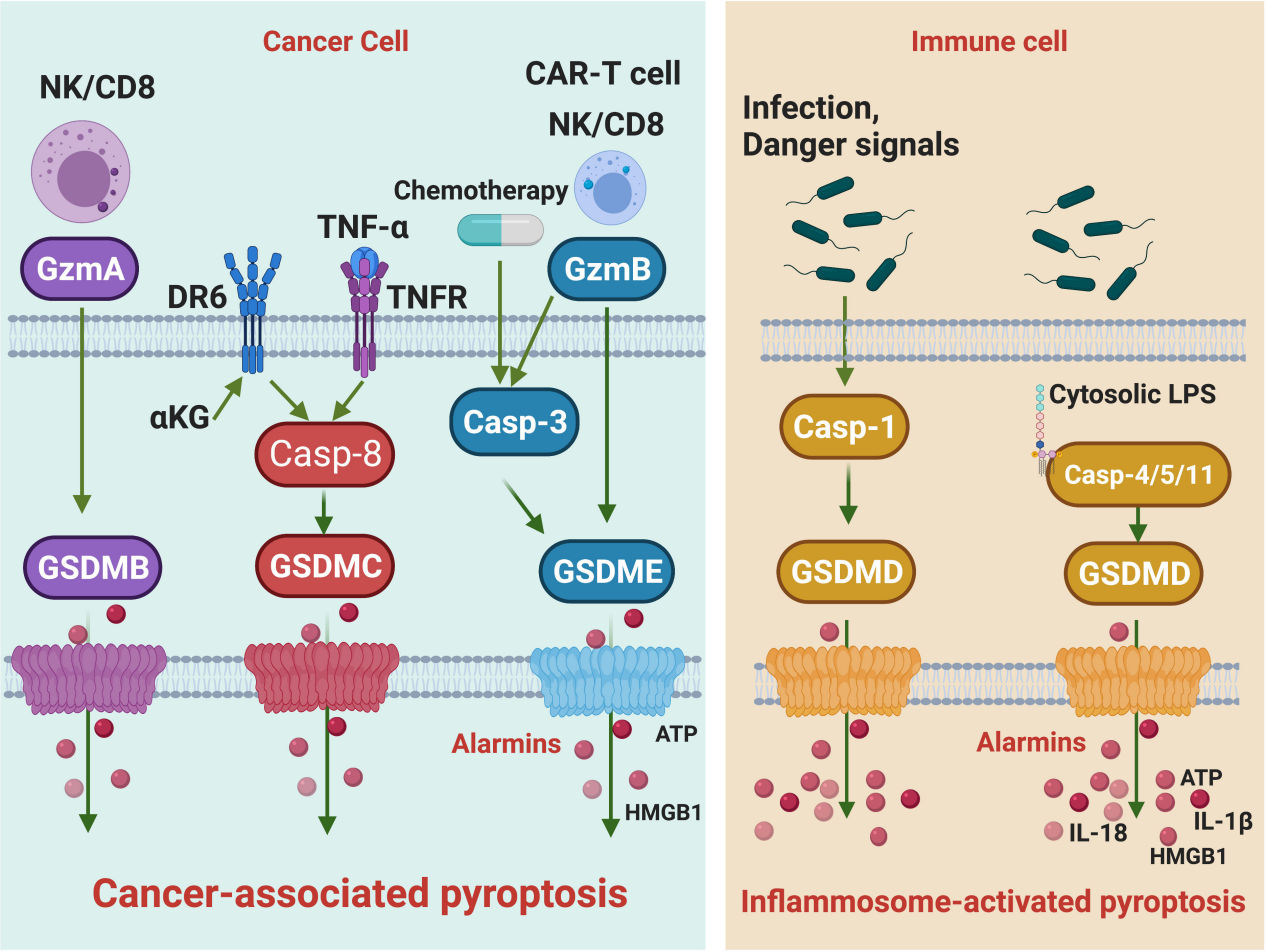
The signaling pathways of cancer-associated pyroptosis and inflammasome-activated pyroptosis. Killer lymphocytes, including NK cells and CD8 T cells, can release Gzms to activate GSDMs and trigger cancer-associated pyroptosis. GzmB cuts GSDME and GzmA cleaves GSDMB. The GzmB-induced pyroptosis can be amplified by caspase-3. In addition, chemotherapy drugs activating caspase-3 can also trigger GSDME pyroptosis. TNF-α and α-KG activate caspase-8 to initiate GSDMC-mediated cancer-associated pyroptosis, respectively. Alarmins such as ATP and HMGB1 are released during cancer-associated pyroptosis. Upon infection or danger signal perception, the assembly of canonical inflammasome leads to caspase-1 activation, which processes GSDMD to trigger inflammasome-activated pyroptosis. In the non-canonical inflammasome pathway, caspase-1/4/5 act as cytosolic receptors for lipopolysaccharide, a conserved component of Gram-negative bacterial cell wall. Recognition of LPS will activate caspase-1/4/5 to cleave GSDMD, leading to inflammasome-activated pyroptosis. Cytokines such as IL18 and IL-1β are released during inflammasome-activated pyroptosis.

The identification of gasdermin family proteins and their pore-forming activities decouples the inflammasome pathways and pyroptosis, which sets the stage for subsequent discoveries of various forms of pyroptosis that are implicated in distinct biological and pathological processes, like cancer. Although GSDMD-mediated IAP has attracted considerable attention, an era of cancer-associated pyroptosis (CAP) mediated by other GSDM proteins is in sight (**Figure 1**). In contrast to IAP, this CAP is independent of inflammasome or caspases. It occurs in tumor cells but not immune cells and is essential in igniting anti-tumor immunity.

### GSDME-mediated CAP

Among the gasdermin family, GSDME, also known as DFNA5, is the most ancient member that first appeared in invertebrate lancelets (57). It was initially identified as a mutated gene in patients with nonsyndromic hearing impairment (58). Mutations of *GSDME* from those patients all result in the skipping of exon 8, which disrupts the autoinhibition and generates an auto-active GSDME protein that causes pyroptosis in cochlear cells (52, 58). GSDME can be cleaved and activated by apoptotic caspase-3, which converts apoptosis to pyroptosis in cells highly expressing GSDME (52), and mediates post-apoptotic secondary necrosis in cells with a low level of GSDME (51). *GSDME* is silenced by promoter hypermethylation (41, 59, 60) and mutated in multiple cancers including gastric, breast, colorectal tumors and melanoma (54), suggesting GSDME could function in tumor suppression (61). Indeed, *GSMDE* expression in breast tumor, colorectal tumor and melanoma suppresses tumor growth and enhances the number and functions of tumor-infiltrated NK and CD8 T cytotoxic lymphocytes (54). This tumor inhibition depends on both infiltrating lymphocytes and the pore-forming activity of GSDME. Mechanistically, NK and CD8 T cytotoxic lymphocytes deliver granzyme B (GzmB) into GSDME-expressing tumor cells, where GzmB cuts GSDME to induce pyroptosis. This process is amplified by caspase-3 because GzmB also activates caspase-3 to cleave GSDME. The resulting pyroptosis further activates killer cell-mediated anti-tumor response, creating a positive feedback anti-tumor loop, leading to tumor suppression (54). GSDME-mediated pyroptosis can be similarly induced by Chimeric Antigen Receptor T (CAR-T) cells (53). In GSDME-expressing melanoma, the combination of BRAF inhibitors and MEK inhibitors treatment, FDA-approved therapy for BRAF V600E/K mutant melanoma, activates caspase-3 and triggers GSDME pyroptosis. This pyroptosis in melanoma cells further increases the number and function of dendritic cells and T cells in tumors, leading to tumor suppression (62).

### GSDMB-mediated CAP

Killer lymphocyte-mediated tumor cell killing is a vital and final step of anti-tumor immune protection. CD8+ cytotoxic T and NK cells eliminate infected or transformed cells by releasing cytotoxic granules, which contain the pore-forming protein perforin (PFN) and cytotoxic proteases called granzymes (Gzms) (63). Gzms, delivered by PFN into target cells, cleave multiple substrates to induce cell death (63). There are 5 human granzymes, including Gzm A, B, M, H and K. GzmA and GzmB are the most abundant and best-studied Gzms. GzmB cleaves after Asp residues like the caspases, and it also cleaves and activates caspase-3, which amplifies GzmB-mediated death (64). All the Gzms independently trigger cell death by different pathways (63, 65–70), which ensures that target cells resistant to any one pathway are killed. Based on those observations, one would speculate that, in addition to the GzmB/GSDME pathway, multiple Gzm pathways could lead to pyroptosis, a safeguard mechanism to ensure that GSDM-expressing target cells resistant to any one pathway are killed by pyroptosis. Indeed, shortly after identifying the GzmB/GSDME pathway, GzmA/GSDMB pathway was also reported to mediate killer cell-triggered pyroptosis (44). GzmA cleaves GSDMB after a major site K244 and a minor site K229. Human GSDMB has no counterpart in mice. However, human GSDMB expression combined with anti-PD-1 treatment significantly suppresses the growth of mouse CT26 colorectal carcinoma and B16 melanoma (44), suggesting that pyroptosis can function synergistically with immune checkpoint blockade. GSDMB expression is mainly detected in the gastrointestinal tract. GSDMB can be induced by interferon gamma (IFN-γ) and tumor necrosis factor alpha (TNF-α), which could be released by tumor-infiltrating CD8 T cells and other immune cells, thus providing a positive feedback loop. Although both GSDMB and GSDME can be activated by killer cells, it seems that GSDME is a more potent tumor suppressor than GSDMB, probably due to the initiation and amplification of GSDME pyroptosis by Caspase-3 in tumors. Whether other Gzms and GSDMs are also implicated in killer cell-triggered pyroptosis awaits further investigation.

### GSDMC-mediated CAP

In addition to killer lymphocytes, cytokines and metabolites can also trigger cancer-associated pyroptosis mediated by GSDMC. A recent study revealed that upon TNF-α treatment, the active caspase-8 cleaves GSDMC, which unleashes the pore-forming GSDMC-NT to trigger pyroptosis in tumor cells (45). Of note, GSDMC normally does not express but can be induced under hypoxic stress through a PD-L1-STAT3 axis. Thus this CAP is closely related to hypoxia and restrained by PD-L1 expression and TNF-α secretion. It was proposed that GSDMC-mediated CAP promotes tumor progression by generating necrosis in tumors. However, most related experiments were performed in nude mice using human and mouse breast cancer cells, excluding the impacts of immune responses. α-Ketoglutarate (α-KG), an essential metabolite in the tricarboxylic acid (TCA) cycle, could also induce GSDMC-mediated pyroptosis in tumor cells. Mechanistically, α-KG induces the oxidation and internalization of the plasma membrane-localized death receptor DR6, which further recruits caspase-8 and GSDMC, leading to caspase-8-mediated GSDMC cleavage and pyroptosis (47). Not like TNF-α, α-KG activates DR6 from within the cells. Treatment of dimethyl-α-ketoglutarate (DM-α-KG), a cell membrane permeable analog of α-KG, suppressed the growth and metastasis of murine B16 melanoma. However, if the tumor suppressive effect of DM-α-KG depends on the immune system has not been determined yet. Further investigation is also required on the activation of GSDMC by caspase-8, because two different cleavage sites, Asp365 and Asp240, have been identified in those two studies, respectively.

### CAP and anti-tumor immunity

Multiple lines of evidence suggest that CAP activates anti-tumor immunity (71). In GSDME-expression tumors, the number and function of dendritic cells, T cells and NK cells are increased (54, 62). More importantly, the tumor suppressive effect of GSMDE depends on those immune cells and is abolished in immune-deficient mice. Of note, GSDME is silenced and mutated in multiple tumors (41, 59, 60, 71), underscoring its essential role in tumor suppression. Moreover, GSDMB expression in tumors significantly increases the efficacy of immune checkpoint blockade (ICB), suggesting pyroptosis can synergize with ICB to promote anti-tumor immunity (44). A recent study has delivered GSDMA3-NT in tumor cells to induce pyroptosis (72). Phenylalanine trifluoroborate (Phe-BF3) is a cancer-imaging probe that can concentrate in tumor cells and desilylate and ‘cleave’ a designed linker that contains a silyl ether. GSDMA3-NT was conjugated by such a linker to the nanoparticle and was delivered and released into tumor cells by Phe-BF3-mediated desilylation. The release of GSDMA3-NT in tumors led to pyroptosis in around 10% of tumor cells, but the whole 4T1 mammary tumor graft was rejected. The tumor rejection was absent in immune-deficient mice or upon T cell depletion, suggesting pyroptosis in tumor cells activates anti-tumor immunity.

### Non-pyroptotic functions of GSDMs

GSDM-mediated cancer-associated pyroptosis, in many cases, play a critical role in activating anti-tumor immunity. However, the pathological roles of GSDMs in cancers seem to be context- and cancer-type-dependent. For instance, high expression of GSDMB correlates with either better or worse survival rates in patients in a cancer-type-dependent manner (44). It is worth noting that GSDM expressions are not simply equal to pyroptosis since GSDMs can also exert non-pyroptotic functions. For example, GSDMB is implicated in epithelial immigration and regulates cell adherence (73). It also promotes tumor cell migration and breast cancer metastasis (74). GSDMD modulates mucin granule secretion in goblet cells and plays an essential role in mucus layer formation (75). GSDMD can also translocate to the nucleus and act as a PARP-1 inhibitor to induce DNA damage and apoptosis in colorectal cancer cells (76). Furthermore, GSDME transports a transcriptional factor YBX1 into the nucleus, which increases mucin expression and promotes the formation of a mucus barrier that prevents chymotrypsin-mediated destruction of pancreatic ductal adenocarcinoma (77). Therefore, the effects of GSDM expressions on tumor growth may be determined by both the pyroptosis and non-pyroptotic functions, which sometimes have opposite impacts on tumorigenesis.

### IL-1β secretion in IAP and CAP

Although both are mediated by GSDM proteins, IAP and CAP are distinct from each other (**Figure 1**): 1) IAP mainly occurs in immune cells, and CAP mainly occurs in cancer cells. 2) IAP is mainly mediated by the inflammasome pathway, and CAP is triggered by various cytosolic proteases. 3) IAP is accompanied by the maturation and secretion of IL-1β (and IL-18), but CAP is not. 4) IAP and CAP may lead to distinct outcomes in tumor growth. The secretion of IL-1β seemly distinguishes IAP from CAP. IL-1β plays dual roles in tumorigenesis (78). It is essential for the establishment of anti-tumor immunity in some contexts. IL-1β produced by NLRP3 IAP in dendritic cells is required to prime IFN-γ–producing CD8 T cells by dying tumor cells (79). Administration of IL-1β increased the population size and functionality of adoptively transferred T cells within the tumor (80). IL-1β is also protective in colitis-associated cancer (81) and myeloma and B-cell lymphoma (82). Nonetheless, it is well documented that IL-1β promotes tumorigenesis. Polymorphisms in the *IL-1β* gene resulting in elevated IL-1β production are associated with an increased risk of gastric cancers (83) and shorter survival in pancreatic cancer patients (84). Chemical carcinogen-induced tumorigenesis is significantly impaired in *IL-1β* knockout mice (85). Indeed, overexpressing human IL-1β in mouse stomachs leads to spontaneous gastric inflammation and cancer, possibly caused by early recruitment of myeloid-derived suppressor cells (MDSCs). Increased IL-1β production is also observed in head and neck squamous cell carcinoma (HNSCC) patients. Pharmacological inhibition of NLRP3 inflammasome in HNSCC mouse models significantly reduces the IL-1β production, which reduces MDSCs, regulatory T cells (Tregs) and tumor-associated macrophages (TAMs) but increases CD4 and CD8 T cells in tumors to improve anti-tumor immunity (86). Therefore, the recruitment of immunosuppressive cells could be a primary mechanism underlying IL-1β-mediated tumorigenesis. IL-1β may also contribute to angiogenesis and invasiveness of the tumor (87–90). The multifaceted roles of IL-1β reflect the roles of IAP and, broadly, inflammation in tumorigenesis. Chronic inflammation, caused by infection, autoimmunity, and environmental or dietary exposure, is believed to promote tumorigenesis. While acute inflammation, induced by therapy drugs or therapy-boosted killer cell attacks, usually enforces anti-tumor immunity and suppresses tumor growth (91). In this regard, IAPs, which are usually activated during infection and autoimmunity, are more likely to generate a pro-tumor chronic inflammatory environment. While CAP, as we discussed before, is mainly initiated by chemotherapy drugs and killer cell attacks that lead to acute inflammation to promote anti-tumor immunity.

Of note, CAP itself does not release IL-1β (no expression and/or activation of inflammasome components in most tumors), but IL-1β production has been observed in tumors with CAP. In CAR-T-cell-treated tumor cells, the robust CAP released a large amount of ATP, which activated NLRP3-mediated inflammasome and pyroptosis in tumor-infiltrating macrophages, leading to the release of cytokines such as IL-1β (53). This IL-1β production is reminiscent of IL-1β secretion in non-canonical inflammasome, which does not generate IL-1β but can trigger secondary NLRP3 inflammasome activation and IL-1β production. When delivering and specifically releasing an active GSDMA3 fragment into tumor cells, GSDMA3-NT similarly triggered CAP as well as IL-1β production. This IL-1β secretion could also be attributed to the secondary activation of IAP (72). Secondary IAP activation and IL-1β secretion may require robust CAP induction, and no IL-1β secretion was reported in other CAPs.

## Harness cancer-associated pyroptosis to ignite anti-tumor immunity

Cancer-associated pyroptosis, rather than immunosilent apoptosis, not only directly kills some susceptible tumor cells, but also elicits robust tumor-specific immune responses, leading to the control or rejection of the entire tumor (54, 72). The role of pyroptosis in anti-tumor immunity makes GSDM proteins attractive therapeutic targets for cancer treatment. Because GSDME could be cleaved and activated by caspase-3, multiple caspase-3-activating chemotherapy drugs have been used to activate GSDME pyroptosis in tumors (92). For instance, BRAF inhibitors plus MEK inhibitors treatment can activate GSDME pyroptosis to enhance anti-tumor immunity in GSDME-expressing melanoma (62). Similar strategies have also been reported for pyroptosis mediated by other GSDMs. As mentioned, α-KG has shown its ability to trigger GSDMC-mediated pyroptosis and suppress tumor growth (47). Serine dipeptidase DPP8/9 inhibitors, which activate GSDMD-mediated pyroptosis in human myeloid cells, induce pyroptosis in most human acute myeloid leukemia cell lines and primary cells but not cells from other lineages, presenting a novel therapeutic strategy for acute myeloid leukemia (93). Multiple nano-technology-based strategies have also been developed to activate pyroptosis in tumor cells (94, 95).

Nonetheless, it is well documented that tumors can evade CAP by suppressing the expression of GSDMs, which hampers the usage of pyroptosis-activating drugs. *GSDME* is suppressed by promoter hypermethylation in gastric, colorectal and breast cancers (41, 59, 60). GSDMA and GSDMB are similarly silenced by methylation in some cancer cell lines (96, 97). DNA methyltransferase inhibitor 5-aza-2’-deoxycytosine (decitabine) upregulates *GSDME* expression and restores pyroptosis in culture cells (52, 60). Decitabine combined with chemotherapy nano-drugs triggers GSDME-mediated pyroptosis and enhances the efficacy of chemotherapy in mice (98). Thus similar strategies might also be used to increase the expressions of other GSDMs in tumors. In addition, some cytokines, chemokines and other signaling molecules could upregulate the expression of GSDMs. For instance, GSDMA is upregulated by transforming growth factor-β (TGF-β) (99), GSDMB by interferons and TNF-α (44), and GSDME by corticosteroid dexamethasone and forskolin (100). It is worth testing whether these GSDM transcriptional modulators could be combined with pyroptosis-inducing drugs to de-repress CAP and potentiate tumor-specific immune responses.

Regardless of endogenous GSDM expressions and activating mechanisms, strategy to directly introduce active GSDM proteins into tumor cells has also been proposed. A recent study described nanoparticle delivery combined with the desilylation-based release of GSDMA3-NT in tumors (72). Phenylalanine trifluoroborate (Phe-BF3) is a cancer-imaging probe that can enter cells to desilylate and ‘cleave’ a designed linker that contains a silyl ether. GSDMA3-NT proteins were conjugated to nanoparticles by a triethylsilyl linker, which can be cleaved by Phe-BF3. Those nanoparticles and Phe-BF3 were both concentrated in tumors, leading to the selective release of GSDMA3-NT in tumor cells to trigger CAP. Interestingly, pyroptosis of around 15% of tumor cells was sufficient to reject the entire transplanted tumor in a T cell-dependent manner, confirming the potential of CAP in activating anti-tumor immunity and eliminating tumors.

While there are great therapeutic benefits of targeting GSDMs and pyroptosis for tumor suppression, the safety of those strategies needs to be carefully evaluated. Uncontrolled and excess activation of pyroptosis may cause severe tissue damage and other adverse effects. DPP8/9 inhibitors, which induce pyroptosis in acute myeloid leukemia, also activate pyroptosis in other cells, such as B cells and CD34+ cells (93) and augment treatment-associated toxicity. Nonspecific activation of GSDME-mediated pyroptosis in normal tissues resulted in extensive tissue damage upon chemotherapy treatments in mice (52). Secondary activation of GSDMD pyroptosis by CAP alarmins in tumor-associated macrophages lies at the root of CAR-T cell-triggered cytokine release syndrome, a severe adverse effect of CAR-T immunotherapy (53). Those observations represent the possible limitations of pyroptosis-based immunotherapeutic strategies. Thus, CAP induction *in vivo* needs to be finely controlled to achieve the specific targeting of tumor cells, which is a major challenge for pyroptosis-based immunotherapeutics.

## Discussion

Identifying GSDMs and their pore-forming activity has revolutionized our understanding of pyroptosis. Pyroptosis has been redefined as a gasdermin-mediated programmed necrotic cell death. In addition to classical inflammasome-activated pyroptosis, recent studies have identified many novel forms of pyroptosis mediated by different GSDMs and activated by various proteases, most of which occur in tumor cells. Pyroptosis in tumors is a highly immunogenic cell death that activates anti-tumor immune protection. Therefore, inducing pyroptosis in tumor cells could have therapeutic utility. Nonetheless, many questions about cancer-associated pyroptosis remain elusive. 1) How does pyroptosis activate anti-tumor immunity? It has been shown that CAP is associated with more active tumor-infiltrating immune cells such as dendritic cells, CD4 and CD8 T cells and NK cells (54, 62), which lead to the control or rejection of the implanted tumors. However, the exact mechanism underlying how pyroptosis recruits and activates those immune cells remains largely unknown. 2) How do cancer cells evade anti-tumor pyroptosis? It is well documented that GSDME is silenced or mutated in multiple tumors (41, 59, 60, 71). However, GSDMB is overexpressed in multiple tumors, and it is controversial if it acts as a pore-forming protein (44, 73, 101). Further investigation is required to clarify if GSDMB-mediated pyroptosis activates anti-tumor immunity and how tumors evade this CAP. 3) How is pyroptosis regulated in cells? The regulation of IAP has been extensively studied (10, 102), but how cancer-associated pyroptosis, mediated by GSDMB, C, and E, is regulated remains elusive and awaits further investigation. 4) How can we harness pyroptosis to ignite anti-tumor immunity to treat tumors, and how can we block pyroptosis-associated toxicity? As we discussed, many strategies have been proposed to employ pyroptosis to kill tumor cells and activate the tumor-specific immune response. How to efficiently and specifically induce pyroptosis in tumor cells is the key to suppressing tumor growth but reducing pyroptosis-associated toxicity. Tumor-targeting GSDM agonists could be a possible way to address the problem.

## Author contributions

ZZ and QK wrote and revised the manuscript. Both authors contributed to the article and approved the submitted version.
